# Is Polyhexamethylene Guanidine and Oligo(2-(2-Ethoxy) Ethoxyethyl Guanidium Chloride Exposure Related to Gestational Diabetes?

**DOI:** 10.3390/toxics12120841

**Published:** 2024-11-22

**Authors:** Hyowon Choi, Nam-Yun Kim, Nalai Kim, Yeon-Soon Ahn

**Affiliations:** 1 Department of Prevention Medicine, Yonsei University Wonju College of Medicine, Wonju 26426, Republic of Korea; wowo0226@gmail.com; 2 Department of Obstetric and Gynecology, Yonsei University Wonju College of Medicine, Wonju 26426, Republic of Korea; rlaskafus493@gmail.com (N.-Y.K.); obgyn.knl@gmail.com (N.K.); 3 Genomic Cohort Institute, Yonsei University Wonju College of Medicine, Wonju 26426, Republic of Korea

**Keywords:** gestational diabetes mellitus, polyhexamethylene guanidine, oligo (2-(2-ethoxy) ethoxyethyl guanidinium) chloride, humidifier disinfectant, mediation analysis

## Abstract

This study aims to investigate the association between exposure to toxic indoor chemicals, specifically polyhexamethylene guanidine (PHMG) and oligo(2-(2-ethoxy) ethoxyethyl guanidinium) chloride (PGH), used in humidifier disinfectants, and gestational diabetes mellitus (GDM). We confirmed pregnancy from 2003 to 2017 and identified GDM by linking a cohort of claimants who reported exposure to PHMG/PGH with National Health Insurance Service data. The GDM incidence was calculated, and PHMG/PGH exposure characteristics—exposure status, the humidifier’s distance/location, and exposure duration/hours—were investigated. Logistic regression and mediation analysis were applied using asthma, frequently treated with steroids, as a mediator. Among 521 pregnancies, 38 were identified as GDM, with 2.4% before exposure and 8.9% after exposure. Pregnancies after exposure had a higher odds ratio (OR) for GDM (OR 2.968, 95% CI: 1.004–12.725). A trend of increased GDM risk was observed with longer exposure duration/hours. Additionally, pregnancies after exposure demonstrated total and direct effects on GDM (β = 0.0435, *p* = 0.036, β = 0.0432, *p* = 0.030) independent of the indirect effects by asthma. The incidence of GDM was higher after PHMG/PGH exposure compared to before. PHMG/PGH exposure was associated with GDM, independent of asthma. Further research is warranted to confirm these findings in exposed cohorts and to explore the underlying mechanisms.

## 1. Introduction

Gestational diabetes mellitus (GDM), the most common pregnancy complication [1], leads to several maternal complications, including an increased risk of developing type 2 diabetes and cardiovascular diseases. Additionally, there may be adverse effects on the child’s health [2]. It typically results from β-cell dysfunction against a background of chronic insulin resistance during pregnancy [2]. Chemical exposures, including endocrine disruptors, fine particulate matter, and organic pollutants, are recognized for damaging pancreatic β-cells and contributing to the development of GDM [3,4]. Humidifier disinfectants (HDs), a class of indoor toxicants, have been associated with severe lung diseases, including HD-associated lung injury and interstitial lung disease (ILD) [5]. HDs were widely used in Korea, particularly among children and pregnant women. According to Chang et al., among 1144 pregnant women who responded to questionnaires, 28.2% of subjects reported using a humidifier [6]. Despite the widespread use of HDs among pregnant women, human epidemiological studies examining the impact of HD exposure on pregnancy complications, including GDM, are lacking.

Recent toxicologic studies suggest that HDs can have systemic effects beyond the lungs, including metabolic disorders and reproductive toxicities [7,8,9]. This mechanism may also be associated with the development of GDM. Also, asthma, a respiratory condition strongly associated with HD exposure [10], is itself a recognized risk factor for GDM due to chronic inflammation or hormonal changes [11]. In addition, asthma is often treated with steroids [12], which are well-known to induce glucose intolerance [13] and further increase the risk of GDM [14]. Given the high prevalence of asthma among individuals exposed to HDs, the relationship between asthma and GDM in this population warrants further investigation. 

Most HD brands contained polyhexamethylene guanidine/oligo(2-(2-ethoxy)ethoxyethyl guanidium chloride(PHMG/PGH) and a mixture of chloromethylisothiazolinone (CMIT) and methylisothiazolinone (MIT) [15]. In in vivo studies with a maximum observation period of 7 days, guanidine-type compounds, such as PHMG and PGH, demonstrated a retention rate of over 66% in the body when inhaled [16]. In contrast, CMIT/MIT compounds showed a retention rate of 22% in the body when inhaled [17]. Furthermore, in vivo studies have reported that PHMG/PGH contributes to pregnancy-induced toxicities [18]. Given these differences, our study focuses on guanidine-type compounds, particularly PHMG and PGH.

In this study, we aimed to (1) estimate the incidence of GDM by PHMG/PGH exposure status; (2) investigate the association between GDM and various exposure characteristics, including pregnancy affected by PHMG/PGH exposure, cumulative exposure duration/hour, and humidifier location/distance; and (3) explore the effect of PHMG/PGH exposure on GDM through both direct pathways and indirect pathways mediated by asthma using mediation analysis based on a causal diagram.

## 2. Materials and Methods

### 2.1. Study Populations

From 2011 to 2020, a total of 7056 claimants registered with the Korean Environmental Industry and Technology Institute (KEITI) for compensation related to damages caused by HDs. The data for these claimants have been systematically documented and stored within KEITI’s comprehensive portal for HD damage support [19]. We selected subjects who reported exposure to PHMG/PGH. By linking this database with the Korean National Health Insurance Service (NHIS) claims data, we tracked the medical utilization of these subjects from 2002 to 2021. Indexes of deliveries were identified using the International Classification of Diseases, 10th Revision (ICD-10), codes O8*, in combination with inpatient records and delivery procedure codes. The conception date for each index delivery was estimated by subtracting 266 days (38 weeks) from the date of delivery, and this duration was defined as the pregnancy episode [20,21]. We selected pregnancy episodes with conception dates between January 2002 and December 2016, which correspond to delivery dates between January 2003 and December 2017, reflecting the five years following the cessation of HD sales.

**Figure 1:**
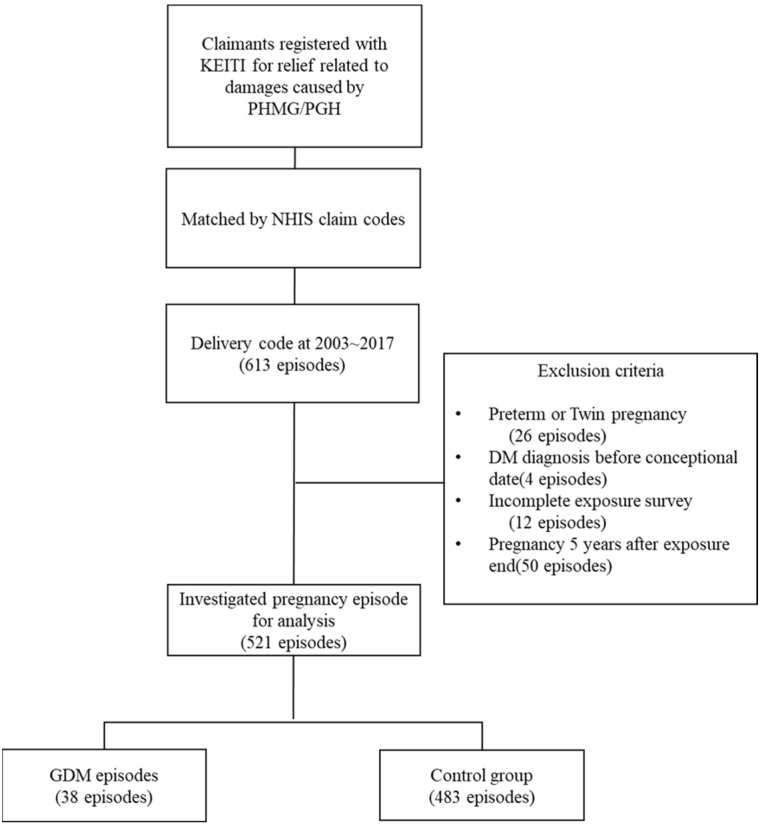
Study flowcharts. KEITI, Korean Environmental Industry and Technology Institute; NHIS, National Health Insurance Services; DM, diabetes mellitus; GDM, gestational diabetes mellitus.

Episodes were excluded if they had twin or preterm deliveries (26 episodes), if they had been diagnosed with diabetes mellitus (DM) prior to conception (4 episodes), if they had incomplete exposure surveys (12 episodes), or if the date of the index delivery was more than five years after exposure cessation (50 episodes), as severe pregnancy outcomes were observed to manifest within a latency period after HD exposure, with a decreasing trend after 2017 [22]. Ultimately, 521 episodes and 374 subjects were included in the analysis (Figure 1).

### 2.2. Outcomes (GDM) and Mediator (Asthma)

In Korea, all pregnant women between 24 and 28 weeks of pregnancy are screened for gestational diabetes mellitus (GDM). From 2003 to 2012, the two-step method was primarily used for GDM diagnosis. However, following the results of the international Hyperglycemia and Adverse Pregnancy Outcome [23], both the one-step and the two-step methods have been utilized since 2013 [24]. As data on the oral glucose tolerance test are not available in the NHIS claims data, GDM was defined as having been diagnosed with the ICD-10 codes O244 and O249, recorded at least twice during pregnancy episodes. 

Asthma was defined as having at least two occurrences based on the ICD-10 codes J45 and J46. It was considered if diagnosed before 28 weeks of pregnancy or prior to the GDM diagnosis.

### 2.3. Assessment of Exposure Characteristic

Exposure data related to PHMG/PGH were obtained from KEITI’s comprehensive portal for HD damage support. The data were collected through a self-administered questionnaire followed by a 1:1 interview with victims who reported damage [19]. Subjects’ exposure was verified using HD receipts and photos of HD purchases.

We classified pregnancy episodes based on PHMG/PGH exposure status as either “after-exposure episodes” if PHMG/PGH exposure began before GDM diagnosis in GDM cases or before 28 weeks of pregnancy in control episodes. For “non-exposure episodes”, we included episodes without any reported PHMG/PGH exposure during the relevant time period.

To identify which exposure characteristics were associated with GDM, we investigated the distance from the humidifier and the humidifier’s location. To estimate the exposure duration, we calculated the cumulative exposure duration. For the GDM, we determined the cumulative exposure duration before the GDM diagnosis. For the control, we determined the cumulative exposure duration before 28 weeks of pregnancy. And, we computed the cumulative exposure time (hours) by multiplying the years of use (year), annual use (months/year), monthly use (weeks/month), daily use (days/week), and hourly use (hours/day). Also, we computed the indoor air concentrations, suggested by Ryu et al. [25], by multiplying the used HD product concentration by the daily average usage amount and dividing by the volume of the space where it was used. For cases where measuring the space volume was not feasible, we substituted with the mean volume from other measured spaces in this study. We then categorized these three variables—cumulative exposure duration, cumulative exposure hours, and concentration—into terciles for analysis.

### 2.4. Covariates and Study Modeling

**Figure 2:**
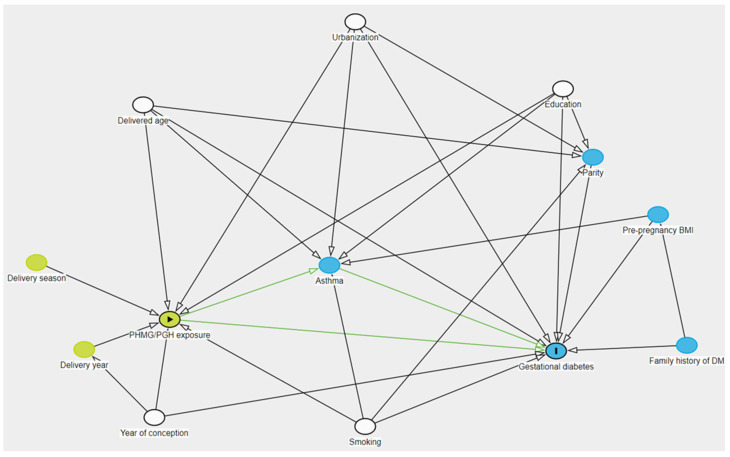
Direct acyclic graph of this study. PHMG/PGH, polyhexamethylene guanidine/oligo(2-(2-ethoxy)ethoxyethyl guanidium chloride; BMI, body mass index; DM, diabetes mellitus; GDM, gestational diabetes mellitus.

Potential confounders were selected using a directed acyclic graph (DAG) to identify variables that could influence the relationship between the main exposure and the outcome, thus reducing bias in the study [26] (Figure 2, Table S1). Known risk factors for GDM, including maternal age at delivery, parity, educational level, smoking status, and urbanization, were considered as confounders [27,28]. The year of conception (≤2013, >2013) was also included due to its correlation with the change in the diagnosis method for GDM [24]. Given the seasonality of humidifier use (prevalent between fall and spring), the season of delivery was categorized into four groups: winter (December to February), spring (March to May), summer (June to August), and fall (September to November).

Educational level was dichotomized into “higher than high school” or not. Smoking status was categorized as either a history of smoking (ex and current smokers) or never smokers. Rural areas were defined based on the Korean residential zoning classification, with regions classified as “eup”, “myeon”, or “ri” considered rural.

### 2.5. Statistical Analysis

Data analysis was conducted using R version 4.4.1 (R Foundation for Statistical Computing, Vienna, Austria). Descriptive statistics (mean ± standard deviation for continuous variables and proportions for categorical variables) were calculated for all episode characteristics. The incidence of GDM (per 100 pregnancies) was estimated and stratified by PHMG/PGH exposure status. Incidence cases were identified as GDM diagnoses, and the incidence rate was calculated by dividing the number of incident cases by the target episodes. We calculated the incidence of GDM by performing age standardization using the age structure (≥30, or <30) of pregnancy episodes in the non-exposure.

Comparative analyses of sociodemographic factors, PHMG/PGH exposure status, exposure characteristics, and comorbidities between GDM and control episodes were conducted using *t*-tests and chi-square tests. Fisher’s exact test was used for variables with a sample size of less than 10. Logistic regression analysis was performed to examine the relationship between PHMG/PGH exposure status/characteristics and GDM.

In the mediation analysis, PHMG/PGH exposure status was considered the exposure, asthma the mediator, and GDM the outcome. All analyses were conducted using both crude and adjusted models. The crude model included only the exposure and the outcome, while the adjusted model included the age at delivery, urbanization, educational level, and smoking status as covariates, based on the DAG. In logistic regression, we adjusted the mediator and the selected confounders. The mediation analysis was performed using the R package “mediation” (version 4.4.7) [29] by employing bootstrapping with 1000 simulations to perform the analysis. Statistical significance was determined at *p* < 0.05 for all analyses.

This study used retrospective cohort data, and there may be unmeasurable variables that may affect causal assumptions. Therefore, we used the same R package to perform the mediation analysis. In the sensitivity analysis, we calculated the correlation (Rho) between the mediator and the outcome variables, as well as the product of the R-squared values between the mediator and the outcome variables (R2M × R2Y). We specified a range of values for Rho and R2M × R2Y, and for each combination of values, we estimated the average causal mediation effect (ACME) using 1000 simulations. Furthermore, due to the limited number of GDM cases, we conducted an additional sensitivity analysis with 1:4 propensity score matching based on selected confounders, followed by conditional logistic regression.

## 3. Results

Table 1 presents the characteristics of the study participants. The participants had a mean birth year of 1976.3 ± 4.4 years, with a total exposure duration of 39.1 ± 32.8 months. Among the participants, 12.3% resided in rural areas, and only 2.4% reported a history of smoking. Additionally, 14.7% had a pre-pregnancy diagnosis of asthma.

The incidence of GDM was 7.3 per 100 pregnancies overall. In non-exposure pregnancy episodes, the incidence of GDM was 2.4 per 100 pregnancies. In pregnancy episodes after PHMG/PGH exposure, the incidence was 8.9 per 100 pregnancies. Based on age standardization of the data using the age structure of women with deliveries in non-exposure, the incidence of GDM was 2.4 per 100 pregnancies in non-exposure cases and 8.2 per 100 pregnancies among after-exposure cases (Table 2).

The characteristics of the study episodes were as follows: the mean age was 31.2 ± 3.8 years, the mean delivery year was 2008.2 ± 3.3, the cumulative total exposure duration was 18.7 ± 23.5 months, the mean cumulative exposure time was 7135.6 ± 11,226.1 h, and the mean concentration of PHMG/PGH was 1.3 ± 2.7 μg/m^3^. A total 4.2% of episodes occurred after 2013, 12.1% of episodes occurred in rural areas, 2.3% of episodes were in ever-smokers, and 28.8% of episodes involved participants with education levels at or below high school. In total, 73.5% of participants reported the humidifier being located “less than 1 m” away, and 73.1% reported the humidifier being positioned “close to the nose and mouth”. The proportion of asthma was 10.6%. A total of 38 episodes (7.3%) were classified as GDM cases. Compared to the control group, significant differences were observed in the age, delivery year, year of conception, exposure status, cumulative exposure hour distribution, and tercile of the concentration of PHMG/PGH (*p* = 0.019, *p* < 0.001, *p* = 0.001, *p* = 0.024, *p* = 0044, and *p* = 0.012, respectively; Table 3).

Table 4 presents the logistic regression results. After-exposure episodes showed increased odds ratios in both crude and adjusted models (crude OR: 4.062, 95% CI: 1.431–17.062; adjusted OR: 2.698, 95% CI: 1.004–12.725). Cumulative exposure duration and cumulative exposure hours were not individually statistically significant. However, the *p* for the trend for cumulative exposure duration was significant (adjusted *p* = 0.009), while the trend for cumulative exposure hours approached significance (adjusted *p* = 0.059). Also, the second tercile value of concentration showed increased odd ratios in both crude and adjusted models (crude OR: 3.6, 95% CI: 1.535~9.118; adjusted OR: 3.059, 95% CI: 1.26~7.956)

**Figure 3:**
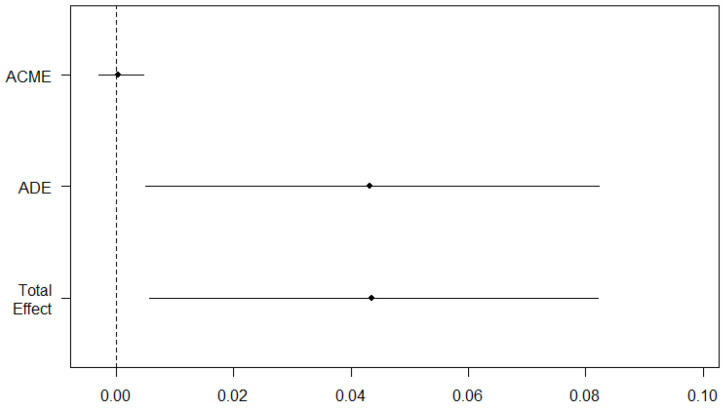
Results of mediation analysis (adjusted model). ACME, average causal mediation effect; ADE, average direct effect.

In the mediation analysis, the crude model showed that the average direct effect (ADE) of exposure status on GDM was statistically significant (β = 0.0650, 95% CI: 0.0276–0.10), as was the total effect (β = 0.0654, 95% CI: 0.0292–0.10). However, the ACME was not significant. In the adjusted model, both the ADE and the total effect remained statistically significant (β = 0.0432, 95% CI: 0.0050–0.08; β = 0.0435, 95% CI: 0.0057–0.08), but the ACME was still not significant (Table 4, Figure 3).

A sensitivity analysis confirmed that the mediation effect of exposure on GDM was insufficient (Table S2). A sensitivity analysis confirmed that the results of the propensity score matching showed no statistically significant values (Tables S3 and S4). However, the direction of the OR values was consistent with the main analysis, and the *p* for the trend for cumulative exposure duration was significant at 0.017.

## 4. Discussion

This study demonstrated a higher incidence of GDM in pregnancy episodes after PHMG/PGH exposure compared to non-exposure episodes, indicating a potential association between PHMG/PGH exposure and GDM. Specifically, increasing cumulative exposure duration and cumulative exposure hours showed a trend toward increasing GDM risk. A moderate exposure concentration also demonstrated a significant association with GDM. Additionally, PHMG/PGH exposure demonstrated a positive total effect and β value for GDM, independent of asthma mediation.

We conducted this analysis using NHIS claim data, which cover 97% of the Korean population and include detailed information about the age, sex, residential regions, medical services, and diagnoses classified through ICD-10 codes [30]. The NHIS claims data, consistently used since 2002, likely captured most GDM cases in our analysis. However, the administrative nature of the NHIS claim data may limit the accuracy of diagnoses. To enhance the accuracy of GDM diagnoses in this study, unlike in other studies [31,32], we additionally confirmed at least two occasions.

A study based on data from the Health Insurance Review and Assessment Service reported that GDM occurred in 7.5% of all pregnancies in Korea between 2009 and 2011 [32]. Similarly, in our study, the incidence was 7.294 per 100 pregnancies, indicating that the analyzed claimants were likely representative of the general population and not subject to selection bias. Nonetheless, when stratified by non-exposure and after-exposure status, we observed a higher incidence of GDM in the after-exposure group, suggesting a potential link between PHMG/PGH exposure and GDM. Furthermore, when the after-exposure group was divided into exposure overlapping pregnancy episodes and pregnancy episodes after exposure had ended, age-standardized data revealed increasing trends in GDM incidence of 6.0% in the exposure overlapping group and 17.1% in the pregnancy episodes after exposure end group (Table S5). Notably, only the pregnancy episodes after exposure ended occurred after 2013, when Korea began using the one-step diagnosis method for GDM alongside the two-step method [24]. According to Chang et al., the incidence of GDM increased from 12.0% in 2013 to 14.9% in 2016, which aligns with the trends observed in our study [33]. Notably, no cases of DM progression were observed among individuals diagnosed with GDM in this study.

In our study, only one episode of ILD was identified, and therefore it was not included as a mediator. Despite the well-established association between ILD and PHMG/PGH exposure, the low incidence observed here may be due to the high risk of adverse pregnancy outcomes—such as miscarriage or preterm birth—when ILD is diagnosed during pregnancy. This could lead to early termination of pregnancies with higher exposure levels, resulting in a potential underrepresentation of high-level PHMG/PGH exposure and ILD cases among full-term pregnant women. Consequently, this may partially explain why the effects of high-concentration exposure are not fully captured in our findings [10].

Our findings also suggest that PHMG/PGH exposure exerts a direct effect on GDM development independent of asthma associated with steroid use and asthma itself. The primary mechanisms driving GDM development involve β-cell dysfunction and chronic insulin resistance during pregnancy [2]. While some chemicals are known to damage or disrupt β-cell function, there is no in vivo study to date directly linking PHMG/PGH to β-cell impairment [4]. However, Ahn et al. reported that women and children exposed to PHMG 5 to 7 years prior had lower serum levels of cholesterol-carrying proteins (APOA4, APOB, APOC1-3, APOE, APOF, APOJ, APOM, and SAA4) and HDL compared to control groups [34]. Low plasma levels of these apolipoproteins and HDL are known to impair lipid metabolism, contributing to insulin resistance [35].

One of the strengths of our study is the comparison of GDM incidence before and after PHMG/PGH exposure, which allowed us to demonstrate the impact of PHMG/PGH on GDM development. Additionally, this is the first study to demonstrate a dose–response relationship and to highlight the direct effect of PHMG/PGH exposure on GDM without asthma mediation. Importantly, we utilized a DAG to guide our analysis, thus ensuring that we avoided over-adjustment, which further strengthens the validity of our findings.

Despite the insights provided by this study, several limitations should be considered. First, we lacked access to data on certain potential confounders, such as pre-pregnancy BMI and family history, which may influence outcomes. However, we confirmed through a DAG that these variables are unlikely to act as confounders in this study. Second, the retrospective nature of the PHMG/PGH exposure data could introduce information bias, although this bias is likely non-differential as it affects both the GDM and control groups equally. Third, our analysis was based on data from individuals who submitted damage claims, which may introduce bias. To mitigate the potential for selection bias from external comparisons, we focused on internal comparisons in this study. Lastly, as this is a retrospective cohort study, it can only establish associations between PHMG/PGH exposure and GDM, not causation. Future experimental studies examining pathophysiology or research with individuals with known exposure histories are necessary to clarify underlying mechanisms.

## 5. Conclusions

In conclusion, this study is the first to demonstrate an association between cumulative PHMG/PGH exposure and GDM, showing that PHMG/PGH directly affects GDM risk without asthma mediation. These findings underscore the need for further research to address potential confounding factors and to conduct mechanistic studies in both animals and humans to further elucidate the GDM risk associated with PHMG/PGH exposure.

## Figures and Tables

**Table 1:** Characteristics of the study participants.

Characteristics	Mean ± SD or n (%)
	(N = 374)
Birth year	1976.3 ± 4.4
Urbanization	
- Urban area	328 (87.7%)
- Rural area	46 (12.3%)
Smoking state	
- Never smoker	365 (97.6%)
- Ever smoker	9 (2.4%)
Educational level	
- >High school	272 (72.7%)
- ≤High school	102 (27.3%)
Total exposure duration	39.1 ± 32.8
Parity	
−1	245 (65.5%)
−2	111 (29.7%)
−3	18 (4.8%)
Asthma diagnosis before GDM diagnosis/G 28 weeks	
- Y	55 (14.7%)
- N	319 (85.3%)

GDM, gestational diabetes mellitus.

**Table 2:** Crude and age standardization incidence of GDM.

Age	Non-Exposure			After Exposure		
	Total Pregnancies, Episodes	Crude Incidence, %	Standardized Incidence, %	Total Pregnancies, Episodes	Crude Incidence, %	Standardized Incidence, %
Total	127	2.36%	2.36%	394	8.9%	8.2%
<30 years	73	1.37%	1.37%	110	7.3%	7.3%
≥30 years	54	3.70%	3.70%	284	9.5%	9.5%

GDM, gestational diabetes mellitus; after exposure began before GDM diagnosis in GDM cases or before 28 weeks of pregnancy in control episodes; non-exposure, not affected by PHMG/PGH exposure.

**Table 3:** Characteristics of study episodes.

N(%) or Mean ± SD	GDM	Control	*p*	Total cases
	(N = 38)	(N = 483)		(N = 521)
Age	32.5 ± 3.4	31.0 ± 3.8	0.019	31.2 ± 3.8
Delivery year	2010.7 ± 3.2	2008.0 ± 3.2	<0.001	2008.2 ± 3.3
Year of conception			0.001	
<2013	32 (84.2%)	467 (96.7%)		499 (95.8%)
≥2013	6 (15.8%)	16 (3.3%)		22 (4.2%)
Delivery seasons			0.837	
Winter (Dec~Feb)	7 (18.4%)	110 (22.8%)		117 (22.5%)
Fall (Sep~Nov)	9 (23.7%)	118 (24.4%)		127 (24.4%)
Spring (Mar~May)	13 (34.2%)	134 (27.7%)		147 (28.2%)
Summer (Jun~Aug)	9 (23.7%)	121 (25.1%)		130 (25.0%)
Parity			0.554	
−1	15 (39.5%)	230 (47.6%)		245 (47.0%)
−2	18 (47.4%)	204 (42.2%)		222 (42.6%)
−3	5 (13.2%)	49 (10.1%)		54 (10.4%)
Urbanization			1	
- Urban area	33 (86.8%)	425 (88.0%)		458 (87.9%)
- Rural area	5 (13.2%)	58 (12.0%)		63 (12.1%)
Smoking state			1	
- Never smoker	37 (97.4%)	472 (97.7%)		509 (97.7%)
- Ever smoker	1 (2.6%)	11 (2.3%)		12 (2.3%)
Educational level			0.835	
- >High school	26 (68.4%)	345 (71.4%)		371 (71.2%)
- ≤High school	12 (31.6%)	138 (28.6%)		150 (28.8%)
Exposure			0.024	
- Non-exposure	3 (7.9%)	124 (25.7%)		127 (24.4%)
- After exposure	35 (92.1%)	359 (74.3%)		394 (75.6%)
Cumulative exposure duration, months (before diagnosis of GDM or G28 weeks)	26.1 ± 25.9	18.2 ± 23.2	0.044	18.7 ± 23.5
Cumulative exposure duration (before diagnosis of GDM or G28 weeks) (tercile)			0.175	
- T1 (≥0, <4)	10 (26.3%)	192 (39.8%)		202 (38.8%)
- T2 (≥4, <20)	11 (28.9%)	138 (28.6%)		149 (28.6%)
- T3 (≥20, <115)	17 (44.7%)	153 (31.7%)		170 (32.6%)
Cumulative exposure hours, hours (before diagnosis of GDM or G28 weeks)	11,687.1 ± 12,757.8	6777.5 ± 11,032.0	0.009	7135.6 ± 11,226.1
Cumulative exposure hours (before diagnosis of GDM or G28 weeks) (tercile)			0.059	
- T1 (≥0, <1008)	7 (18.4%)	170 (35.2%)		177 (34.0%)
- T2 (≥1008, <6720)	13 (34.2%)	161 (33.3%)		174 (33.4%)
- T3 (≥6720, <122,304)	18 (47.4%)	152 (31.5%)		170 (32.6%)
Concentration (μg/m^3^)	2.8 ± 5.5	1.2 ± 2.3	0.088	1.3 ± 2.7
Concentration (μg/m^3^) (tercile)			0.012	
- T1(≥0, <0.001)	8 (21.1%)	207 (42.9%)		215 (41.3%)
- T2 (≥0.001, <1.22)	16 (42.1%)	115 (23.8%)		131 (25.1%)
- T3 (≥1.22, <35.38)	14 (36.8%)	161 (33.3%)		175 (33.6%)
Distance from humidifier			0.401	
- ≤1 m	25 (65.8%)	355 (73.5%)		380 (72.9%)
- >1 m	13 (34.2%)	128 (26.5%)		141 (27.1%)
Location of humidifier			0.665	
- Close to nose or mouth	26 (68.4%)	353 (73.1%)		379 (72.7%)
- Other location	12 (31.6%)	130 (26.9%)		142 (27.3%)
Asthma diagnosis before GDM diagnosis/G28 weeks			0.414	
- Y	6 (15.8%)	49 (10.1%)		55 (10.6%)
- N	32 (84.2%)	434 (89.9%)		466 (89.4%)

GDM, gestational diabetes mellitus; after exposure began before GDM diagnosis in GDM cases or before 28 weeks of pregnancy in control episodes; non-exposure, not affected by PHMG/PGH exposure.

**Table 4:** Logistic regression and mediation analysis by PHMG/PGH exposure characteristics.

Variables	Crude, OR (95% CI)	Adjusted, OR (95% CI)
Exposure state, after exposure, ref. non-exposure	4.03 (1.419~16.926)	2.968 (1.004~12.725)
Cumulative exposure duration (before diagnosis of GDM) (tercile), ref. T1		
- T2	1.53 (0.628~3.77)	1.245 (0.5~3.123)
- T3	2.133 (0.965~4.961)	1.579 (0.681~3.808)
*p* for trend	*p* = 0.024	*p* = 0.056
Cumulative exposure hours (before diagnosis of GDM) (tercile), ref. T1		
- T2	1.961 (0.783~5.333)	1.68 (0.65~4.686)
- T3	2.876 (1.217~7.57)	2.3 (0.929~6.271)
*p* for trend	*p* = 0.009	*p* = 0.017
Concentration (μg/m^3^) (tercile), ref. T1		
- T2	3.6 (1.535~9.118)	3.059 (1.26~7.956)
- T3	2.25 (0.94~5.755)	2.041 (0.844~5.264)
*p* for trend	*p* = 0.663	*p* = 0.681
Distance from humidifier, ref. ≤ 1 m		
- >1 m	1.442 (0.697~2.859)	1.46 (0.689~2.98)
Location of humidifier, ref. close to nose or mouth		
- Other places	1.253 (0.594~2.506)	1.198 (0.555~2.46)
Mediated effects of affected state	Crude, β (95%CI)	Adjusted, β (95%CI)
ACME	0.0004 (−0.0029~0.00)	0.0004 (−0.0031~0.00)
ADE	0.0650 (0.0276~0.10)	0.0432 (0.0050~0.08)
Total effects	0.0654 (0.0292~0.10)	0.0435 (0.0057~0.08)

ACME, average causal mediating effect; ADE, average direct effect.

## Data Availability

The datasets presented in this article are not readily available due to the privacy of the participants. Requests to access the datasets should be directed to Y.-S.A.

## Supplementary Materials

The following supporting information can be downloaded at https://www.mdpi.com/article/10.3390/toxics12120841/s1, Table S1: Code of daggity; Table S2: Sensitivity analysis of mediation analysis; Table S3: Characteristics of study episode after propensity score matching; Table S4: Conditional logistic regression analysis after PSM; Table S5: Crude and age standardization incidence of GDM, further stratified after exposure.
