# Supplementary material for: Is Polyhexamethylene Guanidine and Oligo(2-(2-Ethoxy) Ethoxyethyl Guanidium Chloride Exposure Related to Gestational Diabetes?
Source: Toxics. 2024 Nov 22;12(12):841. doi: 10.3390/toxics12120841 (PMC11679048; doi:10.3390/toxics12120841)
Supplement: Supplementary file 1 [file toxics-12-00841-s001.zip › toxics-3247836-supplementary.pdf]

Table S1. Code of daggity

```
dag {
"Delivered age" [adjusted,pos="-0.845,-0.104"]
"Delivery season" [pos="-0.985,0.251"]
"Delivery year" [pos="-1.006,0.406"]
"Gestational diabetes" [outcome,pos="-0.302,0.450"]
"HD exposure" [exposure,pos="-0.810,0.379"]
"Year of conception" [adjusted,pos="-0.871,0.603"]
Asthma [pos="-0.600,0.257"]
Education [adjusted,pos="-0.293,-0.140"]
Obesity [pos="-0.033,0.256"]
PCOS [pos="-0.083,0.068"]
Smoking [adjusted,pos="-0.553,0.620"]
Urbanization [adjusted,pos="-0.566,-0.291"]
"Delivered age" -> "Gestational diabetes"
"Delivered age" -> "HD exposure"
"Delivered age" -> Asthma
"Delivery season" -> "HD exposure"
"Delivery year" -> "HD exposure"
"HD exposure" -> "Gestational diabetes"
"HD exposure" -> Asthma
"Year of conception" -> "Delivery season"
"Year of conception" -> "Delivery year"
"Year of conception" -> "Gestational diabetes"
"Year of conception" -> "HD exposure"
Asthma -> "Gestational diabetes"
Education -> "Gestational diabetes"
```

```
Education -> "HD exposure"

Education -> Asthma

Obesity -> "Gestational diabetes"

Obesity -> Asthma

PCOS -> "Gestational diabetes"

Smoking -> "Gestational diabetes"

Smoking -> "HD exposure"

Smoking -> Asthma

Urbanization -> "Gestational diabetes"

Urbanization -> "HD exposure"

Urbanization -> Asthma

}
```

Table S2. Plots of Sensitivity analysis of mediation analysis

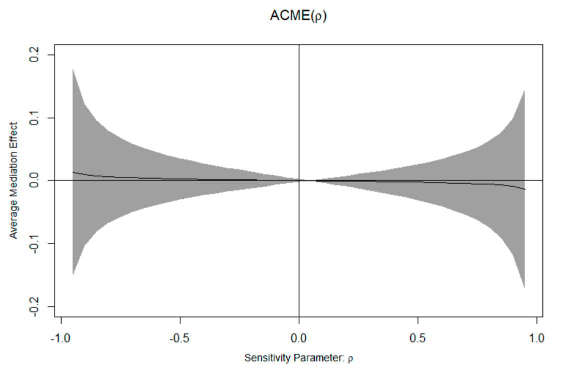	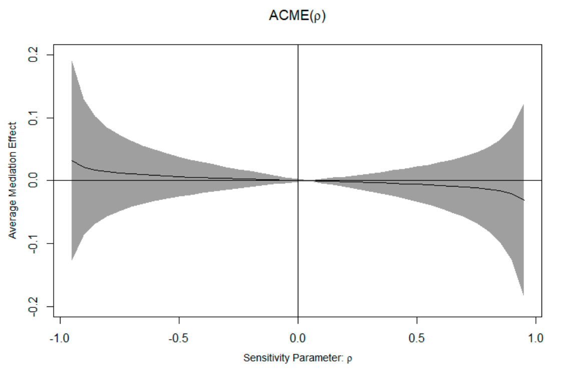
a. Crude model	b. Adjusted model
Rho = 0.005, R^2_M *R^2_Y = 0.0025	Rho = 0.005, R^2_M *R^2_Y = 0.0025

Table S3. Characteristics of Study episode after Propensity score matching

N(%) or Mean ± SD	GDM (N=38)	Matched control (N=152)	p
Age	32.5 ± 3.4	32.6 ± 3.7	0.905
Delivery year	2010.7 ± 3.2	2008.8 ± 3.7	0.004
Year of conception			0.533
< 2013	32 (84.2%)	136 (89.5%)	
≥ 2013	6 (15.8%)	16 (10.5%)	
Delivery seasons			0.955
Winter(Dec~Feb)	9 (23.7%)	32 (21.1%)	
Fall(Sep ~ Nov)	13 (34.2%)	51 (33.6%)	
Spring(Mar~May)	9 (23.7%)	35 (23.0%)	
Summer(Jun~Aug)	7 (18.4%)	34 (22.4%)	
Parity			0.562
-1	15 (39.5%)	74 (48.7%)	
-2	18 (47.4%)	60 (39.5%)	
-3	5 (13.2%)	18 (11.8%)	
Urbanization			1
- Urban area	33 (86.8%)	137 (90.1%)	
- Rural area	5 (13.2%)	15 (9.9%)	
Smoking state			0.477
- Never smoker	37 (97.4%)	150 (98.7%)	
- Ever smoker	1 (2.6%)	2 (1.3%)	
Educational level			0.283
- > High school	26 (68.4%)	92 (60.5%)	
- ≤ High school	12 (31.6%)	60 (39.5%)	
Exposure			0.283
- Non-exposure	3 (7.9%)	25 (16.4%)	
- After-exposure	35 (92.1%)	127 (83.6%)	
Cumulative exposure duration, month (Before diagnosis of GDM or G28weeks)	26.1 ± 25.9	23.2 ± 25.8	0.527
Cumulative exposure duration (Before diagnosis of GDM or G28weeks)(Tertile)			0.857
- T1 (≥0, <4)	10 (26.3%)	47 (30.9%)	
- T2 (≥4, <20)	11 (28.9%)	41 (27.0%)	
- T3 (≥20, <115)	17 (44.7%)	64 (42.1%)	
Cumulative exposure hour, hour (Before diagnosis of GDM or G28weeks)	11687.1 ± 12757.8	7859.2 ± 10572.0	0.057
Cumulative exposure hour (Before diagnosis of GDM or G28weeks)(Tertile)			0.29
- T1 (≥0, < 1008)	7 (18.4%)	41 (27.0%)	
- T2 (≥1008, <6720)	13 (34.2%)	59 (38.8%)	
- T3 (≥6720, <122304)	18 (47.4%)	52 (34.2%)	
Concentration(mg/m3)	2.8 ± 5.5	1.1 ± 1.6	0.08
Concentration(mg/m3)(Tertile)			0.102
- T1(≥0, <0.001)	8 (21.1%)	56 (36.8%	
- T2(≥0.001, <1.22)	16 (42.1%)	41 (27.0%)	
- T3(≥1.22, < 35.38)	14 (36.8%)	55 (36.2%)	
Distance from humidifier			1
- ≤1m	25 (65.8%)	101 (66.4%)	
- > 1m	13 (34.2%)	51 (33.6%)	
Location of humidifier			0.968
- close to nose or mouth	26 (68.4%)	107 (70.4%)	
- other location	12 (31.6%)	45 (29.6%)	
Asthma			0.874
- Y	32 (84.2%)	132 (86.8%)	
- N	6 (15.8%)	20 (13.2%)	

Table S4. Conditional logistic regression analysis after PSM.

Variables	Matched OR (95%CI)
Exposure State, After exposure, Ref. Non-exposure	2.733 (0.705~10.596)
Cumulative exposure duration<br>(Before diagnosis of GDM)(Tertile), Ref. T1	
- T2	1.428 (0.461~4.419)
- T3	2.346 (0.788~6.978)
P for trend	0.017
Cumulative exposure hour<br>(Before diagnosis of GDM)(Tertile), Ref. T1	
- T2	1.386 (0.459~4.183)
- T3	1.355 (0.501~3.668)
P for trend	0.379
Concentration(mg/m3)(Tertile), Ref. T1	
- T2	3.556(1.206~10.483)
- T3	2.133(0.760~5.985)
P for trend	0.708
Distance from humidifier, Ref ≤ 1m	
- > 1m	1.097 (0.509~2.366)
Location of humidifier, Ref. close to nose or mouth	
- Other places	1.030 (0.486~2.180)

Table S5. Crude and Age-standardization Incidence of GDM, further stratified after exposure

	Age	Non-exposure			After-exposure: Exposure overlapping pregnancy episodes			After-exposure: Pregnancy episodes after exposure end		
		Total pregnancies	Crude Prevalence, %	Standardized Prevalence, %	Total pregnancies	Crude Prevalence, %	Standardized Prevalence, %	Total pregnancies	Crude Prevalence, %	Standardized Prevalence, %
All episodes	Total	127	2.4%	2.4%	265	6.4%	6.0%	129	14.0%	17.1%
	<30 years	73	1.4%	1.4%	95	5.3%	5.3%	15	20.0%	20%
	≥30 years	54	3.7%	3.7%	170	7.1%	7.1%	114	13.2%	13.1%
< 2013	Total	127	2.4%	2.4%	265	6.4%	6.0%	107	11.2%	18.4%
	<30 years	73	1.4%	1.4%	95	5.3%	5.3%	12	25.0%	25.0%
	≥30 years	54	3.7%	3.7%	170	7.1%	7.1%	95	9.5%	9.5%
